# COVID-19-Related Restrictions and Quarantine COVID-19: Effects on Cardiovascular and Yo-Yo Test Performance in Professional Soccer Players

**DOI:** 10.3389/fpsyg.2020.589543

**Published:** 2020-12-18

**Authors:** Lucas de Albuquerque Freire, Márcio Tannure, Márcio Sampaio, Maamer Slimani, Hela Znazen, Nicola Luigi Bragazzi, Esteban Aedo-Muñoz, Dany Alexis Sobarzo Soto, Ciro José Brito, Bianca Miarka

**Affiliations:** ^1^Postgraduate Program in Physical Education, School of Physical Education and Sports, Department of Fights, Federal University of Rio de Janeiro, Rio de Janeiro, Brazil; ^2^Brazilian Society of Orthopedics and Traumatology, São Paulo, Brazil; ^3^Postgraduate School of Public Health, Department of Health Sciences (DISSAL), Genoa University, Genoa, Italy; ^4^Department of Physical Education and Sport, College of Education, Taif University, Taif, Saudi Arabia; ^5^Laboratory for Industrial and Applied Mathematics (LIAM), Department of Mathematics and Statistics, York University, Toronto, ON, Canada; ^6^Biomechanics Laboratory, Chilean High Performance Center, Physical Activity, Sport and Health Sciences Laboratory, Universidad de Santiago de Chile, Santiago, Chile; ^7^Escuela de Kinesiologia, Universidad Santo Tomas, Puerto Montt, Chile; ^8^Postgraduate Program in Physical Education, School of Physical Education and Sports, Federal University of Juiz de Fora, Juiz de Fora, Brazil

**Keywords:** athletic performance, behavior analysis, coronavirus, exercise program, physical conditioning

## Abstract

The present study aimed to verify the quarantine’s effects during a serious viral outbreak on the cardiovascular and performance associated with the Yo-Yo test in a sample of professional soccer players. 20 high-level soccer players (*n* = 20; age: 26 ± 4 years-old; weight: 76.85 ± 6.7 kg; height: 179 ± 6 cm) participated in this study. The intermittent Yo-Yo test was performed pre- and post- COVID-19 quarantine in a random order. During each test, the soccer players’ running performance outcomes were monitored using a portable 5-Hz GPS with a 100 Hz accelerometer and a paired *t*-test was conducted at a *p*-value of ≤ 0.05. The main results demonstrated significant differences between pre- versus post-COVID-19 quarantine in the following variables: relative distance (161.7 ± 5.9 > 141.1 ± 33.8 m/min), maximal speed (18.7 ± 0.9 > 18.2 ± 0.6 km/h), acceleration (60 ± 20 frequency > 52 ± 16 frequency), deceleration (34 ± 13 frequency > 27 ± 6 frequency), sprints > 19 km/h [0.8 (0.2;3)% >0.5 (0;0.5)%], and in high intensity running distance [16.48 (2.68;41.64)m > 0.827 (0.164;3.0)m]. We concluded that COVID-19-related restrictions and quarantine COVID-19 demonstrated adverse effects on professional soccer players’ Yo-Yo tests performance.

## Introduction

“Coronavirus disease 2019” (COVID-19) is a new, rapidly emerging zoonotic infectious disease (World Health Organization [WHO], 2020). The first case was reported from the city of Wuhan (Hubei Province, Mainland China) on 31 December 2019. On 30 January 2020, the World Health Organization (WHO) declared the outbreak as a public health emergency of international concern, and, on 11 March 2020, as a global pandemic (World Health Organization [WHO], 2020). COVID-19 is caused by a novel coronavirus, named “Severe Acute Respiratory Syndrome Coronavirus type 2” (SARS-CoV-2), which is transmitted via droplets during close unprotected contact with an infector and fomites (World Health Organization [WHO], 2020). Currently, no effective pharmacological interventions or vaccines are available to treat or prevent COVID-19. For this reason, behavioral, non-pharmacological public health measures such as isolation, physical distancing, quarantine, and even lockdown of entire communities and territories are the only effective ways to respond to the outbreak (World Health Organization [WHO], 2020).

Besides its social, psychological, and economic impact, COVID-19 has affected sports activities too, with major competitions and events being canceled/postponed (Fuentes-García et al., 2020; Guerrero-Calderón, 2020). Also, professional soccer has been affected (Guerrero-Calderón, 2020). Every year, soccer clubs invest significant amounts of money in professional players, with soccer being one of the most popular and competitive Olympic sports worldwide. Therefore, this sport was one of the first to return to training and competition practices, even during quarantine, but the cardio-respiratory condition of the players after this isolation period has not been investigated yet.

Cardiovascular fitness is one of the most important aspects of physical fitness in soccer (Castagna et al., 2017; Slimani and Nikolaidis, 2019). During professional competitions, athletes dispute between two and five games per month, with each game lasting for ∼90 min, a time span, that, in case of a tie, can be extended (Castagna et al., 2010; Costa et al., 2019). Moreover, athletes have training sessions twice a day during the competitive season and take part in national and international championships (Slimani et al., 2018b). This hard and demanding training schedule and tournaments involve great developed physical, mental, and physiological factors (Slimani et al., 2018a; Degens et al., 2019). A well-developed aerobic fitness is essential for soccer players to maintain repetitive high-intensity actions within a soccer match, to accelerate the recovery process, and to maintain their physical condition at an optimum level during the entire season (Orntoft et al., 2016; Michailidis et al., 2020).

Currently, the Yo-Yo intermittent test is the most adequate test for aerobic fitness assessment, with different versions and levels for soccer athletes (Castagna et al., 2020b). The reason for its acceptance is associated with its logical validity (i.e., comprising intermittent high-intensity protocols) (Gimenez et al., 2019), with practical application for testing a large number of players at the same moment with practically no related costs (Povoas et al., 2016; Castagna et al., 2019, 2020b; Papanikolaou et al., 2019). As such, the Yo-Yo intermittent tests are the most explored field tests for aerobic fitness valuation, and are crucial for evaluating soccer athletes (Oberacker et al., 2012; Beato et al., 2019; Castillo et al., 2019).

It is well documented that Yo-Yo intermittent test performance was affected by many factors, such as the detraining period and the expertise level of players (Joo, 2016). However, literature reports contrasting findings regarding this aspect. Short-term (1 and 2 weeks) detraining appears not to have any significant effects on Yo-Yo intermittent test in professional soccer players (Joo, 2016; Rodríguez-Marroyo et al., 2018). In contrast, other studies found that the short-term detraining period (2 weeks) decreased performance in the Yo-Yo test (Thomassen et al., 2010; Christensen et al., 2011; Joo, 2018). This contradiction may be due to the expertise level of players (Joo, 2018).

In the scholarly literature, there is a dearth of data concerning the effects of physical distancing in athletes after COVID-19 related quarantine and restrictions: for the above-mentioned practical reasons, field-testing represents an interesting approach for exploring such effects. Indeed, no published paper has addressed so far the external responsiveness (i.e., sensitivity) of the Yo-Yo intermittent tests in tracking professional players’ VO_2_max development after a period of physical distancing, quarantine, and potential detraining (i.e., pre-to-post-COVID-19 physical distancing and related restrictions; Castillo et al., 2019; Povoas et al., 2020). Therefore, the aim of the present study was to verify the effects of the quarantine during a serious viral outbreak on the cardiovascular and performance associated with Yo-Yo test in a sample of professional soccer players.

## Materials and Methods

### Participants

From an initial list of 82 state professional soccer players of different line positions (30% Midfielders, 12% Defenders, 20% Wingers, and 38% Strikers), the final sample was randomly composed by 20 professional soccer players assessed in two different moments, pre- COVID-19 quarantine (*n* = 20; age: 26 ± 4 years-old; weight: 75.9 ± 6.9 kg; height: 179 ± 6 cm, and Body Fat: 13.8 ± 2.9%) and post- COVID-19 quarantine (*n* = 20; age: 26 ± 4 years-old; weight: 76.85 ± 6.7 kg; height: 179 ± 6 cm, and; Body Fat: 14.6 ± 3.75%). These athletes were from professional soccer teams (Brazil League Serie A) and competed in national and international representative championships once (∼90 min) per week and had been regularly training 2 h of technical and tactical aspects 4–7 times a week, 1 h of physical preparation 2–3 times a week, before the quarantine. During the quarantine of 40 days, they conducted three times a week for 30 min per session of aerobic training between ∼65 and∼75% of maximal heart rate. More specifically, they performed:

• 10 min of warm-up workout and cool-down;
• 15 min of mini-band workouts: 3× the 60 s each exercise in the continuous circuit training: jumping jacks, jumping squat tap, alternating forward lunges, burpees, fast lateral walks;
• 15 min of functional exercises: 8 min dynamic mobility workout – 30 s skipping with and 30 s without shoulder rotation, 30 s skipping with high knees, 30 s running with butt kicks, 30 s side to side with and 30 s without arm movement, 30 s running opening the gate and 30 s closing the gate, 240 s running with leg stretch and swing variations 1:10 s, 2 min of suicide drills and 5 min coordination with the ball;
• 15 min exercise bike workout.

Pacing frequency during workouts and functional exercises were controlled per athletes, using their heart rate. In addition to the heart rate, athletes were familiarized with the Borg’s RPE-scale (6–20; Borg, 1985) and instructed not to exceed efforts ∼14 (McArdle et al., 2014).

Athletes were periodically monitored by direct and indirect cardiovascular tests. Inclusion criteria were the following: being aged over 18 years, without cognitive alterations, without surgeries or injuries, and with more than 3 years in the professional soccer level, and presenting COVID-19 symptoms. Asymptomatic cases or discharged ones were considered eligible. Exclusion criteria were the following: being unable to carry out the Yo-Yo test or suffering from limitations during the study, mainly for health reasons, duly certified by doctors. Besides this, the participants were instructed not to intake alcohol or drugs for at least 24 h before the measures and were maintaining regular diets.

Before proceeding with data collection, all participants attended a briefing meeting and signed a written, informed consent document to understand the testing parameters and the risks and benefits of the study. In addition, a letter of consent was sent and duly signed by all the participants’ soccer clubs. This study was submitted to and approved by the Local Committee of Ethics in Research, following the National Health Council’s rules of resolution and according to the World Medical Association (WMA) Declaration of Helsinki.

### Procedures and Measures

The Yo-Yo intermittent test was performed in a random order. All tests were realized at the same time of day, avoiding circadian effects on test performance (Castagna et al., 2020b). A doctor was monitoring the Yo-Yo test. A standardized warm-up was done before the test, consisting of 10 min of 20 m back and forth running at an intensity subjectively set by the participants (2–3 of the Börg CR10 scale) and with changes of direction of 180° to mimic the evaluation protocols, preceding each Yo-Yo intermittent test (Castagna et al., 2020b). Participants were asked to progressively increase the intensity toward the end of the warm-up, nonetheless without reaching maximal speed. A 2-min passive recovery preceded each test (Castagna et al., 2020b). On the day before testing, participants refrained from performing vigorous physical activity (Castagna et al., 2020b).

The Yo-Yo Intermittent Endurance Test Level 1 (initial speed: 10 km final speed: 19 km) was designed to evaluate the ability to perform intense exercise repeatedly during a prolonged intermittent exercise (Krustrup et al., 2015). In the test, each participant performed a series of 20-m shuttle runs at a pace set by an audio metronome from a calibrated CD player (Sony CFD-V7), with a standard resting interval between shuttles (5 s; Bradley et al., 2014). The time allowed for the shuttles was progressively decreased, while the speed was increased (Lago-Penas et al., 2014). The test was terminated when the subjects failed twice to reach the starting line, or the participant felt unable to complete another shuttle at the dictated speed (Castagna et al., 2020b). The VO_2_ max (ml/kg/min) was assessed as indicated in Povoas et al. (2020) study.

During each test, the soccer players’ running performances were monitored using a portable 5-Hz GPS unit (Catapult, Melbourne, Australia) with a 100-Hz accelerometer. The GPS device was positioned via an elasticized shoulder harness to sit between the scapulae of the bowler at the base of the cervical spine (Petersen et al., 2009). The GPS unit was then activated and the GPS satellite lock was established for at least 15 min before the player taking the field as per the manufacturer’s recommendations (Petersen et al., 2009). After each session, the recorded information was downloaded using Catapult Sprint software (Catapult Innovations, Melbourne, Australia) for analysis, as used by preceding protocols (Beenham et al., 2017). The mean number of satellites and the horizontal dilution of position were recorded during data collection (Abbott et al., 2018). Horizontal dilution of precision (HDOP) designates accuracy of GPS in a horizontal plane (Catapult Sports), and optimal satellite availability (HDOP = 0.0) is where one satellite is directly overhead with a minimum of four spaced equally around the horizon. During Yo-Yo test, HDOP ranged from 0.8–1.6, which is considered a good signal (Catapult Sports). External loads of GPS parameters followed a standardized protocol (Abbott et al., 2018) and are described in Table 1.

**TABLE 1:** Description of external loads parameters.

**External loads**	**Description**
Total distance (m)	Distance traveled during all the test
Relative ditance (m/min)	Distance traveled during all the test per min
Sprints > 19 km/h (%)	% distance traveled running > 19 km/h
Total of sprints (freq.)	Number of sprints > 19 km/h
High intensity runningdistance (m)	Distance traveled during the test > 19 km/h
Max speed (km/h)	Maximum speed during the test
Number of Acceleration (freq.)	Number of accelerations > 2 mss
Number of decelerations (freq)	Number of decelerations > 2 mss
Change direction to the right	Number of change direction to the right
Change of direction to the left	Number of change direction to the left
Explosive efforts (freq)	Sum of accelerations and deceleration
Maximum Heart Rate (bpm)	Maximum heart beats per minute
Walking or jogging distance	Distance traveled during the test with 0 > 11 km/h
Moderate speed running distance	Distance traveled during the test with 11 > 15.5 km/h
Fast speed running distance	Distance traveled during the test with 15.5 > 19 km/h
Total time (min)	Total time in the test

### Statistical Analysis

The Kolmogorov-Smirnov test (K-S) was used to determine the normal distribution of data. The null hypothesis was rejected for sprints > 19 km/h (%; *p* = 0.011) and in the high intensity running distance (*p* = 0.008). For parametric data, descriptive analysis was performed and computed as mean (X) and standard deviation (±SD). In contrast, non-parametric data were described as median (1st quartile – Q1; 3rd quartile – Q3). A paired *t*-test (parametric data) and the Wilcoxon test (non-parametric data) were conducted to compare two moments (pre- versus post-COVID-19 quarantine). The significance level was set at *p* < 0.05 for all analyses. Subsequently, the effect size measure for non-parametric analysis was calculated, defined as ES = Z/√N, where ES represents the effect size, *Z* is derived from the *z*-score of the Wilcoxon (W) test and *N* is the total number of observations. This analysis considers ES-values as small effect size (*r* = 0.10), medium effect size (*r* = 0.30), or large effect size (*r* = 0.50). Data were analyzed using the “Statistical Package for the Social Sciences” (SPSS v. 22.0 program, SPSS, Inc., Chicago, IL, United States).

## Results

Table 2 shows the findings of the descriptive analysis of pre- versus post-COVID-19 quarantine.

**TABLE 2:** Descriptive analysis of yo-yo test analysis in two moments, pre versus post quarantine.

**Variables**	**Mom.**	**Mean**	**SD**	**95% C.I. of the Diff.**		***t***	***p*-value**
				**Lower**	**Upper**		
Total distance (m)	Pre	1570.51	409.93	−275.78	90.45	−1.068	0.301
	Post	1477.84	367.43				
Relative distance (m/min)	Pre	**161.74**	**5.90**	−**37.46**	−**3.79**	−**2.585**	**0.019**
	Post	**141.11**	**33.79**				
Total of sprints (freq.)	Pre	0.28	0.96	−0.75	0.20	−1.230	0.236
	Post	0.00	0.00				
Max speed (km/h)	Pre	**18.72**	**0.93**	−**0.99**	−**0.09**	−**2.538**	**0.021**
	Post	**18.18**	**0.61**				
Acceleration (freq.)	Pre	**59.94**	**19.89**	−**15.02**	−**0.87**	−**2.368**	**0.030**
	Post	**52.00**	**15.86**				
Decelerations (freq.)	Pre	**33.67**	**12.62**	−**13.41**	**0.07**	−**2.086**	**0.05**
	Post	**27.00**	**6.13**				
Change to the right	Pre	18.67	10.30	−7.38	3.05	−0.877	0.393
	Post	16.50	9.40				
Change to the left	Pre	20.67	15.35	−6.69	1.47	−1.351	0.194
	Post	18.06	14.55				
Explosive Efforts (freq.)	Pre	9.00	8.72	−5.42	2.42	−0.808	0.430
	Post	7.50	6.04				
Walking/jogging (m)	Pre	226.36	57.49	−3.93	60.12	1.851	0.082
	Post	254.45	56.87				
Moderate running speed (m)	Pre	471.28	117.93	−109.58	11.32	−1.715	0.105
	Post	422.15	89.49				
Fast speed running (m)	Pre	871.83	256.15	−173.01	35.86	−1.385	0.184
	Post	803.26	242.80				
Total time (min)	Pre	9.65	2.38	−0.48	3.05	1.542	0.142
	Post	10.94	2.92				
Variables	Mom.	Median	Q1;Q3	Effect size		*w*	*p*-value
sprints > 19 km/h(%)	Pre	0.83	0.16;3.0	0.3922		660.50	0.006
	Post	0.52	0.0;0.5				
High intensity running distance (m)	Pre	16.48	2.68;41.24	0.3849		656.50	0.008
	Post	0.94	0.0;7.1				

*Bold values represents significant differences between pre- and post-quarantine values, p > 0.05.*

Statistical analysis indicated a significant reduction of ∼12.5% in relative distance, 13.3% in acceleration and 19.8% in deceleration during quarantine, with an impact on the maximal speed performed (*p* ≤ 0.05 for all comparisons). The Wilcoxon test demonstrated significant differences in sprints > 19 km/h and in the high-intensity running distance with a medium effect size.

## Discussion

The present study aimed to verify the effects of the COVID-19 induced quarantine on the cardiovascular and performance associated with Yo-Yo test in professional soccer players. Our results indicated a significant main effect on the relative distance, maximal speed, acceleration, and deceleration. To the best of the authors’ knowledge, this is the first article that observed COVID-19-related restrictions and quarantine COVID-19 induced effects on cardiovascular and Yo-Yo test performance in professional soccer players. In the present investigation, maximal speed, sprints > 19 km/h, high-intensity running distance, acceleration and deceleration in Yo-Yo test had a significant effect between pre- and post-COVID-19 quarantine. Quarantine and self-isolation were two public health measures that could prevent, or at least minimize, the impact of infectious disease outbreaks in professional soccer teams. However, some professional players still got sick. In addition to this problem, our results could have been influenced by changes in lifestyles and nutritional habits. Despite this, the reduction of physical activity did not contribute to weight gain during the quarantine.

Previous reports indicated that straight line sprinting is categorized as acceleration, deceleration and maximal running speed (Haugen et al., 2014). Yo-Yo test has a higher ecological validity and reliability (Castagna et al., 2019; Francini et al., 2019), since soccer matches performance analyses have shown that >90% of all sprints in games are shorter than 20 m, acceleration and deceleration capabilities are essential for soccer athletes (Modric et al., 2019). Still, the importance of peak velocity increases during the game when sprints start from a jogging or non-stationary condition (Datson et al., 2014; Palucci Vieira et al., 2019; Slimani and Nikolaidis, 2019), with the Yo-Yo intermittent test being useful to evaluate the physical fitness condition of soccer players in terms of levels (Redkva et al., 2018; Povoas et al., 2020; Castagna et al., 2020b), gender (Emmonds et al., 2019; Castagna et al., 2020a), and age classes (Ehlert et al., 2019; Francini et al., 2019).

Concerning our sample, even with a normalized and controlled oxygen saturation between 96 and 98% during the COVID-19 quarantine, the players showed a significant pre- versus post-variation in the results. Our outcomes indicated a significant impact during determinant situations (maximal speed, sprints > 19 km/h, high-intensity running distance, acceleration and deceleration). Moreover, an adaptive planning process to return to the optimum levels occurred. Coaches, analysts, and physiologists played a substantial role in athletes’ physical capacities, using their expertise to evaluate the athlete and providing insight into how effective a post-COVID-19 training system could be improved to increase the athlete’s performance. If the training system was not optimal, then the performance enhancement team reevaluated and modified the design. All athletes received daily medical attention during this initial evaluation and training, as well as psychological support for any emotional problems.

Match analyses indicated that outfield soccer players cover 9 to 12 km during the game, ∼10% of this amount is higher than 19 km/h (Rampinini et al., 2010; Rogers et al., 2019). The present decrease in the relative distance, maximal speed, acceleration, and deceleration of soccer athletes could be associated with the lack of sprint training, and reduction of the total training load during the COVID-19 quarantine. Therefore, present results indicated that quarantine without specific or general stimulus had a negative effect on determinant high-intensity actions. Practical applications of these results suggest realizing sprint intervention on soccer players without COVID-19 contagious to maintain short sprint abilities. Previous reports indicated that short training with distances < 30 m improves specific maximal speed during the games (Randers et al., 2010; Nyberg et al., 2016; Hostrup et al., 2019).

**FIGURE 1:**
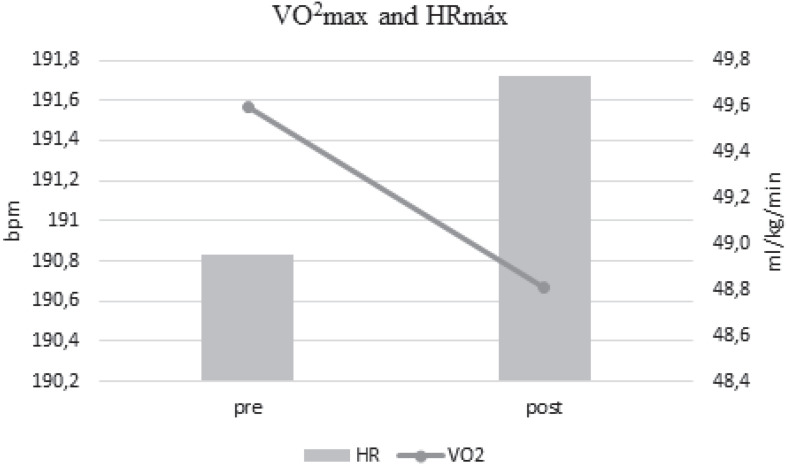
VO_2_ max and HRmax pre- and post-quarantine.

Present results agree with previous reports about VO_2_max values of soccer athletes, ranging between 48 and 62 ml/kg/min for soccer players (Slimani et al., 2019). The typical competitive soccer season has from 8 to 9 months, with a mean of two matches a week and a high aerobic intensity demand estimated at ∼80% of VO_2_max by the game (Slimani et al., 2019). No effects were observed in VO_2_max of Yo-Yo test comparing between pre- and post-COVID-19 quarantine in the present study (Figure 1). The low level of VO_2_max value can explain this before the quarantine period. Coaches and conditioning specialists should include/recommend a specific training program targeting VO_2_max performance during the COVID-19 quarantine, off-season and competitive season to improve the cardiovascular condition in soccer players. In addition, our results highlight the focus on sprinting training progression and periodization/timing, as the sprint is the most frequent mechanism related to hamstring injuries, as ∼20% of all injuries in soccer are hamstring damages (Duhig et al., 2016).

Concerning Yo-Yo test test-retest reliability, investigation indicated intra-class correlation coefficients (ICCs) for test–retest reliability ranging from 0.78 to 0.98, with ∼60% of all ICCs0 > 0.90, while ∼95% of ICCs were 0 > 0.80 (Krustrup et al., 2003; Castagna et al., 2012; Deprez et al., 2014; Povoas et al., 2016; Ehlert et al., 2019). Moreover, the present study controlled for several factors, including those which may affect Yo-Yo test performance, as caffeine ingestion (Ranchordas et al., 2018), participants maintained their usual nutritional habits during all the moments of the collected data (Chtourou et al., 2011), restricting any caffeine intake. Past research indicated that time of day could affect the exercise performance; our measures were standardized between 4 and 6 P.M. (Rampinini et al., 2010; Buchheit and Rabbani, 2014) and all athletes slept between 8 and 10 h in the night before the testing sessions (Fernandes et al., 2015; Fowler et al., 2015).

Although the results of VO_2_max did not demonstrate significant effects of COVID-19 quarantine, coaches and athletes without coronavirus should focus on speed and explosive strength during this moment. In a periodized approach to training whereby training phases with specific adaptive responses have to be appropriately sequenced, following guidelines recommendations, at least 200–400 min of aerobic exercise/week (Fallon, 2020; Jimenez-Pavon et al., 2020) and two times of resistance training/week resistance training performed during COVID-19 quarantine seems to be appropriate (Fallon, 2020; Jimenez-Pavon et al., 2020) – for soccer athletes, our results indicated a distinct focus on specific muscular strength, agility, and power of specific motor skills.

## Conclusion

Our findings demonstrated that COVID-19 related restrictions and quarantine had adverse effects on increasing, decreasing, or maintaining sprints of professional soccer players during the Yo-Yo test. In contrast, aerobic training at 65–75% of maximal heart rate maintained the aerobic capacity during the quarantine and could be a protective health intensity. A multi-component soccer-training program could be considered as the most adequate for professional athletes, including aerobic, resistance, balance, coordination, and specific power motor abilities with short running actions, accelerations, and decelerations.

## Data Availability Statement

The raw data supporting the conclusions of this article will be made available by the authors, without undue reservation.

## Ethics Statement

The studies involving human participants were reviewed and approved by before proceeding with data collection, all participants attended a briefing meeting and signed a written, informed consent document to ensure the understanding of the testing parameters and the risks and benefits associated with the study. In addition, a letter of consent was sent and duly signed by all the soccer clubs of the participants. This study was submitted to and approved by the Local Committee of Ethics in Research, following the rules of resolution of the National Health Council and according with the World Medical Association (WMA) Declaration of Helsinki. The patients/participants provided their written informed consent to participate in this study.

## Author Contributions

LA, MT, MSa, and BM conceived and conducted the experiment, wrote the manuscript, analyzed data, and wrote the manuscript. EA-M, DS, MSl, HZ, BM, and CB revised the manuscript. All authors contributed to the article and approved the submitted version.

## Conflict of Interest

The authors declare that the research was conducted in the absence of any commercial or financial relationships that could be construed as a potential conflict of interest.
